# An Intrinsic Fiber-Optic Single Loop Micro-Displacement Sensor

**DOI:** 10.3390/s120100415

**Published:** 2012-01-04

**Authors:** Alejandro Martinez-Rios, David Monzon-Hernandez, Ismael Torres-Gomez, Guillermo Salceda-Delgado

**Affiliations:** Centro de Investigaciones en Optica, Loma del Bosque 115, Col. Lomas del Campestre, Leon, Guanajuato, 37150, Mexico; E-Mails: dmonzon@cio.mx (D.M.-H.); itorres@cio.mx (I.T.-G.); salceda@cio.mx (G.S.-D.)

**Keywords:** fiber sensors, displacement sensor, tapered fiber, fiber loop

## Abstract

A micro-displacement sensor consisting of a fiber-loop made with a tapered fiber is reported. The sensor operation is based on the interaction between the fundamental cladding mode propagating through the taper waist and higher order cladding modes excited when the taper is deformed to form a loop. As a result, a transmission spectrum with several notches is observed, where the notch wavelength resonances shift as a function of the loop diameter. The loop diameter is varied by the spatial displacement of one end of the fiber-loop attached to a linear translation stage. In a displacement range of 3.125 mm the maximum wavelength shift is 360.93 nm, with 0.116 nm/μm sensitivity. By using a 1,280 nm broadband low-power LED source and a single Ge-photodetector in a power transmission sensor setup, a sensitivity in the order of 2.7 nW/μm is obtained in ∼1 mm range. The proposed sensor is easy to implement and has a plenty of room to improve its performance.

## 1. Introduction

Many industrial and scientific processes require measurement of micro-displacements. Optical fiber sensors have been applied with success in measurements of micro- and nano-displacements offering advantages such as immunity to electromagnetic interference, chemical passivity, stability, multiplexing possibilities and repeatability. We may distinguish two types of fiber optic displacement sensors: extrinsic sensors, where the fiber merely transports the signal from the object being displaced as in reflective [1–4] or interferometric [5–7] sensors; and intrinsic sensors, where the fiber sensitive element experiences displacement, as in those based on fiber Bragg gratings [8,9], or long-period fiber gratings [10]. In general, in most intrinsic fiber sensors the displacement produces a deformation which bends the sensible portion of the fiber, thus affecting the optical transmission properties of the fiber. The sensible portion of the fiber may consist of fiber Bragg gratings [8,9], long-period fiber gratings [10], two-core fibers [11], single mode fibers with high bend loss at the measurement wavelength [12], tapered fibers [13–15], among others. In reference [11], an intrinsic micro-displacement sensor based on a two core fiber is proposed, where the sensitivity depends on the bending deformation suffered by multiple fiber loops. In [12] an ultrasensitive intrinsic fiber sensor based on the macrobending loss at 1.55 μm in a single-mode fiber with wavelength cutoff around 1,060 nm was demonstrated. In this case the measurement range was in the order of 250 μm and a maximum resolution in the order of 40 nm is claimed. In the last two cases the fiber is *per se* sensitive to the bending caused by the displacement. Another simpler alternative for displacement sensors using tapered fibers under bending has been proposed [13–15]. In Reference [13] the bending angle is taken as a measure of displacement which is used as an indirect method to implement acoustic, magnetic, and electric sensors. In this case, the bending is applied directly to the taper at the center of the waist by using a capillary tube, which may perturb the interaction between modes at the waist of the taper. It would be advantageous to avoid the direct perturbation of the interaction zone, so that a given sensor application relies on pure bending. A possible solution would be bending the taper by fixing one or both ends to a linear stage that bends the fiber as the fixing points are moved in the inward direction. However, from time to time small transverse variations may cause deviation in the direction towards the taper while is bent. Another solution would be to form a loop with the taper, and use the perturbation of the total bend determined by the loop radius as a measure of displacement.

In this work, we demonstrate a sensor based on a single-loop fiber taper, where the displacement is measured by analyzing the dependence of the optical fiber transmitted signal on the radius of the tapered fiber loop. At a given radius, the spectral notches formed are shifted with the loop radius change reaching up to 360.93 nm of wavelength shift in 3.125 mm of displacement range. The proposed sensor also can be interrogated by changes in the transmission intensity or by dual wavelength referencing. Using a low power LED source and a Ge photodetector we demonstrate a maximum sensor sensitivity of 2.7 nW/μm, which assuming 10 nW resolution in the photodetector this means that we may detect displacements in the range of 3.7 μm. Using a monocromatic higher power light source and a higher order sensitivity photodetection system we may significantly increase the resolution of our device.

## 2. Principle of the Single-Loop Fiber Displacement Sensor

**Figure 1.**
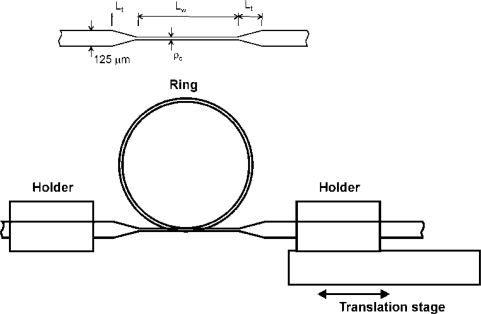
Sketch of the single-loop fiber optic displacement sensor.

In Figure 1 we show a sketch of the steps for the implementation of the proposed displacement sensor. First, a taper is fabricated in standard SMF-28 fiber using a Vytran glass processor system. In Figure 1(a) we show the structure of the fiber tapers used, in which we can distinguish two taper transitions, with a length L_t_, situated at both sides of a constant diameter cylindrical section, known as taper waist with a length *L_w_* and a diameter of ρ_w_. In this study we used three distinct tapers with a *L_t_* of 3, 3, and 1.5 mm, and a *ρ_w_* of 40, 35 and 40 μm, respectively. In all cases the length of the waist was 30 mm. Other waist diameters were also tested, however, for higher waist diameters the fiber taper loop radius needed to detect spectral changes induced high enough stress to break the fiber, while for smaller waist diameters no sensitivity at all was detected with the proposed setup. Once the taper was fabricated, one fiber end was attached to a fixed fixture, then the taper waist was deformed to form a loop, and the free fiber end was fixed to a fixture attached to a linear translation stage. Before fixing the taper end to the translation stage, a small twist was applied in order to overcome the static repulsion by the resultant shear force at the touching zone where the loop is closed. The shear force induced by the twist is applied in a perpendicular direction with respect to the linear translation that changes the loop radius, so that it does not perturb significantly its movement. As the loop radius is changed by the linear translation, a change in the transmission spectrum is observed, with several spectral notches that shift according to the radius variation.

At the taper waist, the V-parameter of the light propagating through the core is V_core_∼0.6913 at a wavelength of 1.55 μm, so that the light from the fundamental core mode is transferred to the fundamental cladding mode [13,14]. The V-parameter for the cladding mode is determined by the cladding-air interface and has a value of V_c_∼167 meaning that it can support the propagation of thousands of modes. When the fiber taper is deformed to form a loop with its waist, and the loop radius is changed, the curvature radius modifies the effective refractive index profile of the tapered fiber, which can be modeled as an equivalent straight fiber with refractive index profile n_e_ given by [16]:

(1) $$n_{e}=n(r)\;{\left(1+\frac{2\;r}{R_{c}}\text{cos}(\phi )\right)}^{1/2}$$

where *n*(*r*) is the refractive index profile of the straight fiber, *R_c_* is the curvature radius of the bend, and, *r* and *ϕ* are the local radial and azimuthal coordinates, respectively. As a result of the refractive index asymmetry induced by the bend, the fundamental cladding mode excites other cladding modes. In fact, the bending also causes an increase in the cutoff value of the V-parameter (in core and cladding), so that with bending, the condition *V_core_* < *V_c_* is clearly satisfied [17]. Obviously, the type and number of excited modes depends on the curvature and hence can be used as a measure of displacement by the proper design of the varying curvature radius mechanism.

**Figure 2.**
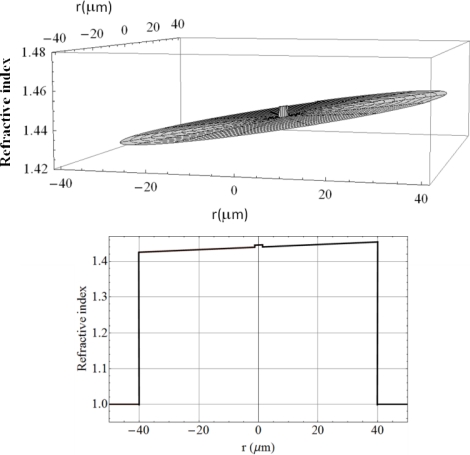
Refractive index distribution at the fiber bent assuming a curvature radius of 4.77 mm.

The upper graph in Figure 2 shows a revolution plot of the refractive index profile and lower graph shows a cut of the transverse refractive index profile for *ϕ* = 0, assuming a curvature radius of 4.77 mm, *i.e.*, the curvature radius corresponding to a circle with a perimeter equal to the waist length.

It is clearly seen that the refractive index profile at the edge of the cladding-air interface is higher than that of the core and depends on the curvature radius as given by relation (1). The asymmetry and magnitude of the refractive index at the cladding edge, depending on the curvature radius, determine the cladding modes to be excited and hence the transmission properties of the loop. In other words, the effect of the curvature is to break the rotational symmetry of the fiber, allowing the coupling of the fundamental mode with even and odd cladding modes. In principle, the strongest coupling will be with the modes that have the propagation constant closest to the fundamental cladding mode [18], however this requires to be verified by numerical calculations. Another effect that affects the transmission characteristics is the modal mismatch at the transition between the curved and straight waveguide, where in the first case the modal distribution shifts towards the outer boundary, while in the second case the modes are symmetrically centered thus resulting in scattering loss [19]. In Figure 4 of reference [18] the calculation of the effective indices of the fundamental and the second mode as a function of the bending radius is shown. As mentioned in [18], as the curvature radius increases, the propagation constants of the fundamental and higher order modes start to differ significantly thus reducing the coupling. Since the range of curvature radius where our device operates is between 3.3–4.77 mm, we may expect from [18] that we are in the zone where significant coupling is expected between the fundamental and higher order modes. It is worth to note that in our sensor (Figure 1) the inter-fiber coupling at the touching point that close the loop is very small even for waist diameters of the order of 8.5 μm [20], so that the proposed device does not work as a loop resonator but as a bending based device. We found that the bending is easily controlled by varying the loop radius and we did not observe any noticeable hysteresis in going from one direction to the other. In fact the tapers used to form the loops reported in this work were adiabatic, with less than 0.3 dB insertion loss at the straight position, and showed high bending sensitivity with others bending setups, for example by fixing the taper ends at two linear stages that moves inward and outward to control the bend, or by S-bend where one of the fixtures is displaced transversally with respect to the other. Although in the last two cases the insertion loss caused by bending were lower than in the case of the fiber loop, small movements in the transverse direction resulted in spectral or power transmission changes as high as those due to the linear displacements, particularly with the S-bend setup. This effect may be useful for a 3D displacement sensor, but requires a lengthy characterization to map the sensor response. It is worth to mention besides the standard single mode fiber SMF-28 used here, other fibers such as DS/SMF28 and HP980 were tapered and used to form the loops. In all cases a similar response was observed, however here we only show the results obtained with SMF28 fiber.

## 3. Spectral Characterization of the Fiber-Loop Displacement Sensor

**Figure 3.**
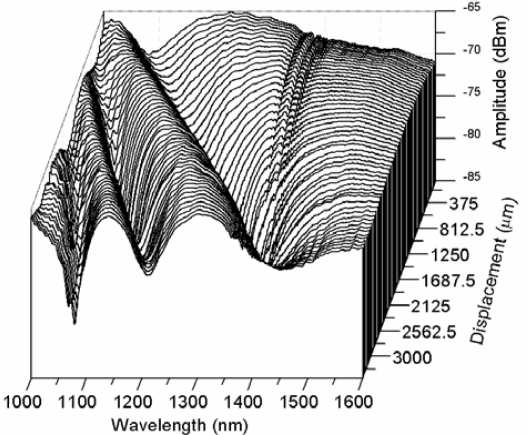
Waterfall plot of the spectral evolution of the fiber-loop displacement sensor.

**Figure 4.**
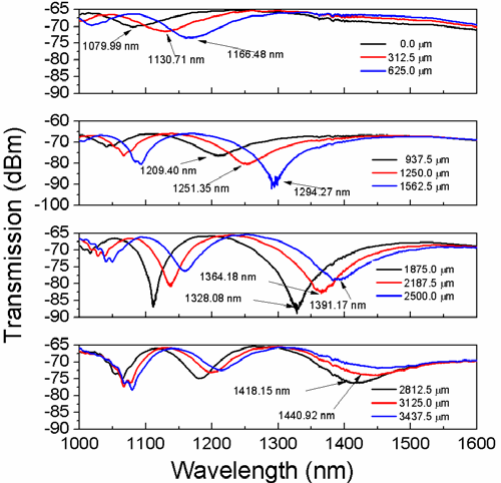
Spectral evolution of the transmission spectrum from the zero position (3.89 mm loop radius) to a displacement of 3,437.5 μm.

We fabricated several fiber tapers that were mounted and tested in the experimental set-up represented in Figure 1. As the fiber loop diameter changes, due to the displacement of the translation stage, the coupling between the fundamental and higher order cladding modes produces a change in the transmitted spectrum. Figure 3 shows a waterfall plot of the spectral evolution of the fiber-loop displacement sensor taken at 62.5 μm displacement steps in a decreasing curvature radius direction. In this case the waist has 30 mm length and 40 μm diameter. At the zero displacement position (taken at 3.89 mm loop radius) there is a spectral notch that shifts towards longer wavelengths in ∼3.4 mm displacement range. At displacement distances of ∼0.625 and ∼2.5 mm a second and third notches appear, that also experience a wavelength shift toward longer wavelengths as the displacement distance increases. A closer look into the spectral evolution can be seen in Figure 4.

**Figure 5.**
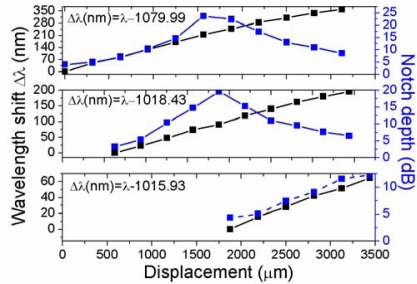
Spectral shift and notch depth as a function of displacement distance for the three major notches observed in Figure 4.

Figure 4 shows the spectral evolution from 0 to 3,437.5 μm of displacement in steps of 312.5 μm. At the zero position there is a notch at ∼1,079.99 nm with a depth of 3.981 dB (solid black line in the upper graph) that shifts to longer wavelengths as the displacement is increased; meanwhile its depth decreases until it practically disappears for a 3,437.5 μm displacement. A second and third notches appear at 1,018.43 nm (3.316 dB depth) and 1,015.93 nm (4.356 dB depth) when the displacement distance is 625 μm (solid blue line in the upper graph) and 1,562 μm (solid blue line in the second graph from the top to the bottom), respectively. In all cases the central wavelength of the spectral notches shifts to longer wavelengths as the displacement distance is increased (*i.e.*, in the direction of decreasing loop radius). As it can be observed, as the notches shift to longer wavelengths there is a variation in their depth. The upper graph in Figure 5 shows the evolution of the spectral notch with the larger displacement range (3.125 mm) and higher wavelength shift (360.93 nm). This spectral notch reaches a maximum depth of 23.77 dB (blue curve in upper graph of Figure 5) at 214.28 nm of wavelength shift, and has a sensitivity of 0.116 nm/μm in the whole range. Assuming that we can resolve spectral shifts in the order of 0.1 nm, then the displacement resolution is on the order of 0.86 μm. The second graph from the top to the bottom in Figure 5 shows the evolution of the next notch with the larger displacement range, where a maximum wavelength shift of 195.52 nm is observed in a displacement range of 2.8125 mm. In this case the maximum notch depth is reached at 91.14 nm wavelength shift, and the sensitivity is ∼0.07 nm/μm which for 0.1 nm spectral resolution means that the sensitivity to displacement is ∼1.4 μm. The bottom graph in Figure 5 shows the evolution of the spectral notch with the smaller displacement range (1.562 mm) and wavelength shift (64.71 nm) and hence the lowest sensitivity (∼0.04 nm/μm), with a displacement resolution in the order of 2.5 μm for a spectral resolution of 0.1 nm.

From Figures 4 and 5 we observe that there is a strong variation in the spectral transmission with displacement which can be used to monitor either the displacement of a certain notch or the intensity variation at one or several wavelengths. A way to evaluate the variation in the transmitted power as a function of displacement is by taking the area under a given spectral range. This is equivalent to assume that the photodetection system has a flat spectral response in the measured range.

**Figure 6.**
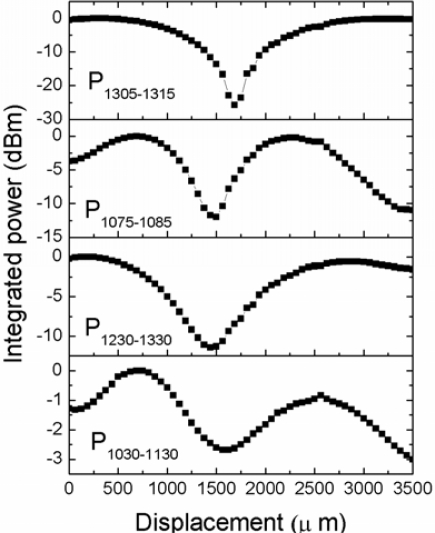
Integrated spectral power as a function of displacement at several spectral ranges.

Referring to Figure 6 from the bottom to the top, we see that a broad light source from 1,030–1,130 nm (like that available from a LED source) only produces changes in the transmitted power (P_1030–1130_) as high as 2.67 dBm, while with a broad light source emitting in a 1,230–1,330 nm range the maximum change in the transmitted power (P_1230–1330_) may be as high as 11.28 dBm.

**Figure 7.**
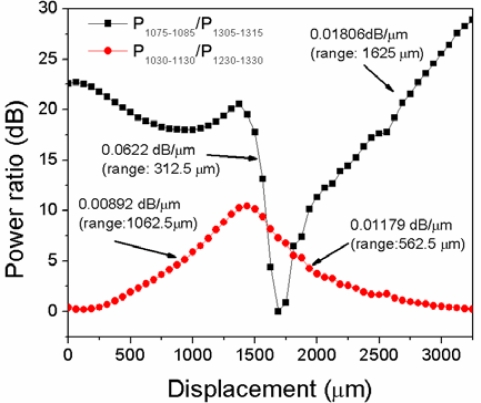
Ratio between integrated spectral powers as a function of displacement.

On the other hand, in principle, the use of a narrowband light sources should increase the resolution of the power transmission measurements. In the third and fourth graphs of Figure 6 the integrated power from 1,075–1,085 nm (P1075–1085) and from 1,305–1,315 nm (P1305–1315), respectively, which may correspond to laser light sources with a 10 nm bandwidth, is shown. As can be seen, in the lower wavelength range the maximum change in the transmitted power has increased from 2.67 dBm to 11.97 dBm, while in the higher wavelength range the maximum change increased from 11.97 dBm to 25.7 dBm. It is noteworthy that the useful displacement range in a real application will be in the region where the change in the transmitted power shows a pronounced slope, which by inspection of Figure 6 it is on the order of ∼1 mm. Another possibility for the sensor interrogation is by the use of a dual wavelength referencing method [21], where the ratios between the power at two different spectral ranges are evaluated to increase sensitivity. To evaluate the performance of the displacement sensor in a dual wavelength referencing setup, we take the ratio of the integrated spectral power in two different wavelength regions. The red circle points in Figure 7 show the dB ratio between the integrated spectral power from 1,030–1,130 nm (P_1030–1130_) and the integrated power from 1,230–1,330 nm (P_1230–1330_) nm in the displacement range from 0–3,500 μm, while the black square points show the ratio between P_1075–1085_ and P_1305–1315_. The labels at the tails of each curve indicate the slope and the displacement range that was used to calculate it. It can be seen that the highest displacement sensitivity (0.0622 dB/μm) is found at a displacement range of only 325 μm for the ratio P_1075–1085_/P_1305–1315_, which, assuming that 0.05 dB of ratio change can be resolved, means that the displacement resolution will be in the order of 0.8 μm. On the other hand, the highest displacement range (1.625 mm) is also found for the ratio P_1075–1085_/P_1305–1315_. In this case, assuming 0.05 dB of ratio change resolution, the displacement resolution will be ∼2.5 μm, which still has practical applications.

## 4. Intensity Based Displacement Sensor

**Figure 8.**
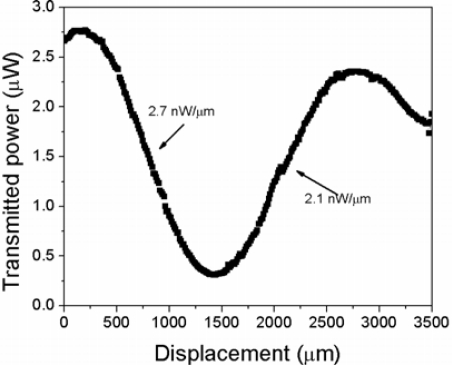
Measured transmitted power as a function of displacement by using a 1,280 nm, 100 nm bandwidth LED source and a Ge photodetector.

Figure 8 shows the measured transmission power as a function of displacement. In this case, a low power broadband LED source with 1,280 center wavelength and 100 nm bandwidth was used as the excitation source, and the output power was measured using a Ge photodetector. Comparing this result with that obtained in the second graph from the bottom to the top in Figure 6, we see that there is a good correlation between both curves, since the maximum power variation in the first case is ∼9.45 dBm, and 11.97 dBm in the second case, while the position of the maximum change in both cases is around 1.5 mm of displacement. The small difference is due to the non-flat spectral photodetector response in the spectral range of the excitation source. In addition, the LED excitation source has a Gaussian-like spectral distribution around its central wavelength of 1,280 nm. The labels close to the lobes edges in Figure 8 show the slope of the linear regions at each slope, which in both cases span ∼1 mm displacement range. Taking the slope as a measure of the sensitivity to displacement, we observe that the highest sensitivity is 2.7 nW/μm in a displacement range around 1 mm. If we assume that the photodetector can resolve power changes around 10 nW, the resultant displacement resolution will be around 3.7 μm.

To evaluate the effect of the transition length of the taper used to form loop in the sensor performance we used a taper with 1.5 mm transitions length and 30 mm waist length in the same type of fiber (SMF28). In this case we only measured the maximum measured sensitivity, which was 1.361 nW/μm, which assuming a 10 nW resolution of the photodetector, means that the loop sensor in this case can resolve displacements in the order of 10 μm.

**Figure 9.**
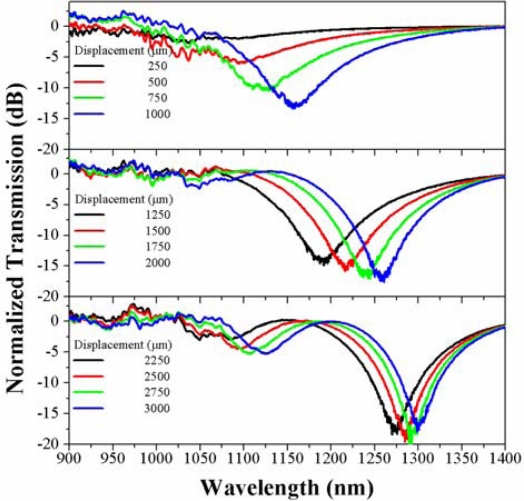
Spectral evolution of a loop displacement sensor made with a 3 mm transition length, 30 mm waist length, and 35 μm waist diameter of taper, in a displacement range from 0–3 mm.

**Figure 10.**
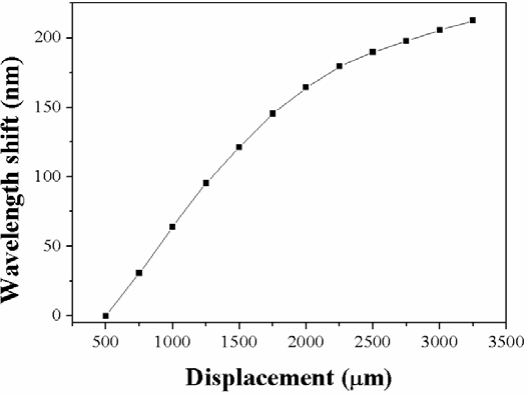
Wavelength shift as a function displacement distance for a loop displacement sensor made with a 3 mm transition length, 30 mm waist length, and 35 μm waist diameter of taper.

On the other hand, keeping the same transition and waist lengths (3 mm–30 mm–3 mm) but decreasing the waist diameter to 35 μm, we observe a spectral behavior similar to the case of a taper with 40 μm waist diameter. Figure 9 shows the spectral evolution in a displacement range of 3 mm. In general we observe that the notch experiences a shift toward longer wavelengths as we increase the displacement distance. In this case the maximum displacement observed is in the order of 212 nm (Figure 10).

**Figure 11.**
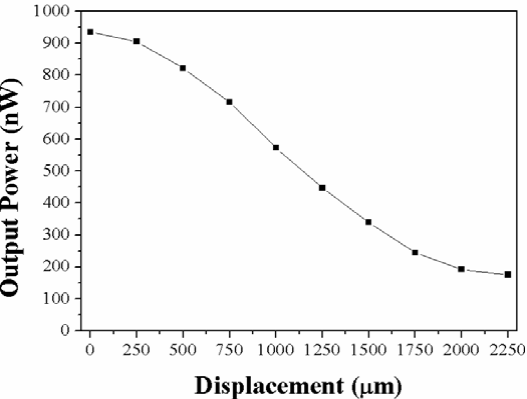
Output transmitted power as a function of displacement distance for a loop displacement sensor made with a 3 mm transition length, 30 mm waist length, and 35 μm waist diameter of taper.

For this particular sensor, intensity based measurements by using the broadband LED source and the Ge photodetector resulted in a sensitivity of 0.45 nW/μm (Figure 11), which means that we may resolve displacements in the order of 22 μm, which is still useful for many industrial applications.

**Figure 12.**
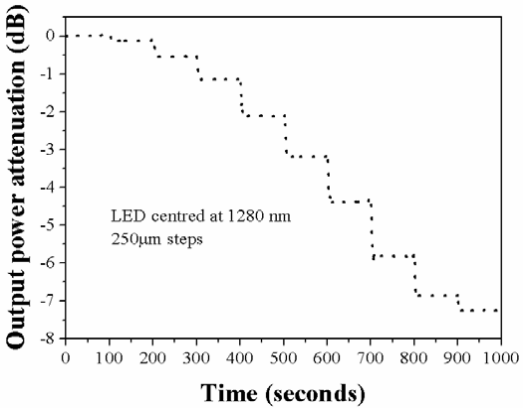
Time stability of the fiber loop displacement sensor.

The time stability of the sensor was measured by keeping the fiber loop displacement sensor at a given position by 100 s. Figure 12 shows the result of such measurement, where the measurements were made in steps of 250 μm. For this measurement we did not use any vibration damping mechanism for the optical table and the air conditioning was on, so that even in the presence of that kind of ambient perturbations the device is clearly stable in time. Besides the stability, simplicity, low cost, and possibility to interrogate the device by several means, the intrinsic character of our sensor is a great advantage since by a proper fixture design, we may attach it to any machinery or apparatus even in the toughest environmental conditions.

It is well known that temperature variations affect the performance of any optical fiber sensor [12]. When temperature changes there are thermo-optic and thermal expansion effects that may modify the power transmission characteristics which may manifest as an undesirable attenuation in the whole spectral range or the wavelength shift of a given spectral notch. This effect is particularly important in ultrahigh resolution measurements. In order to evaluate the effect of temperature in our proposed device we enclose the zone of the fiber loop in a box made of thermal insulation material. Inside the insulating box there was an electrical heat source just below the fiber loop, and a thermocouple just above it. Power transmission measurements were made at three different temperatures (22 °C, 35 °C, and 44 °C) in a 3 mm displacement range. In this displacement range, the fiber loop used in this measurement shows a monotonic behavior.

**Figure 13.**
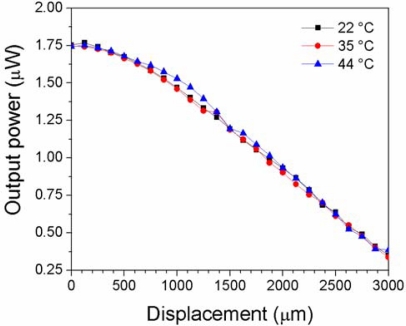
Output transmitted power as a function of displacement distance for a loop displacement sensor made with a 3 mm transition length, 30 mm waist length, and 35 μm waist diameter of taper at three different temperatures.

Figure 13 shows a graph of the transmitted power as a function of displacement at the three different temperatures. It can be observed that the effect caused by the temperature variation is not appreciable since it is obscured by power variations of the optical excitation source, in this case a low power broadband LED source. This observation coincides with a measurement made by using a white light source, where we observed that at a given position the increase of temperature from 25 °C to 36 °C resulted in ∼0.1 dB loss. A more accurate measurement will require the use of a setup where the optical power source variations are compensated such as that proposed in reference [12].

## 5. Discussion

Several questions arise about the operation of the proposed fiber loop displacement sensor proposed in this work. A close view to the spectral evolution of the transmitted spectrum tell us that as the displacement is increased the spectral notches shift to longer wavelengths reaching a maximum depth in the region where the input not tapered fiber supports more than one mode. After this maximum, the notch depth starts to decrease until it eventually disappears at the region where the input not tapered fiber is strictly single-mode. This behavior may be explained as a manifestation of the existence of two modes at the input of the taper, which become the fundamental and first higher order modes of the cladding at the taper waist. As it was mentioned already, these two modes have the closest propagation constants for the bending radius range in which our device is operating. As the bend radius (or loop diameter) is changed, the wavelength for maximum coupling between these modes, moves to the longer wavelength side. When we move farther from the cutoff wavelength of the input non-tapered fiber, only the fundamental core mode enters into the taper. Then the coupling strength between the fundamental and the first higher order cladding modes at the waist decreases. This may explain the spectral behavior observed in Figures 4 and 9. Another question to be answered is why do we select 40 μm as the waist diameter and not another size. As was demonstrated experimentally in Section 4, when the waist diameter is reduced to 35 μm the sensitivity to displacement of the proposed sensor decreases considerably. If we decrease the waist diameter, say to 20 μm, no spectral changes are observed at all. This observation coincides with the theoretical findings of reference [18], where in the bending range where our device is operating the difference between the propagation constants of the fundamental and first higher order cladding mode at the taper waist is considerably, thus reducing their coupling strength. On the other hand, when a wider waist diameter is used, in the bending range where the loop is sensitive, the stress induced by the bend is strong enough to break the fiber. Thus, a waist diameter around 40 μm seems to be the optimum for this particular application.

On the other hand, the analysis of the experimental results in Sections 3 and 4 allow us to assert that the device sensitivity in power transmission measurements may be significantly increased by the use of narrow band light sources. Also, it is possible to use higher sensitivity photodetectors or schemes to reduce the noise fluctuations that may significantly increase the resolution of our device. Since, the bending results in a waveguide birefringence (see Figure 2), we may push the resolution of our device to the limit by the use of narrowband, polarized light sources in conjunction with polarimetric detection systems. In future, we will be working on the exploration of such schemes to improve the device sensitivity. Thus, in principle the fiber loop displacement sensor proposed in this work has the capability to measure displacements in the order of micrometers or less, which due to its simplicity and intrinsic character has applications in several industrial processes.

## 6. Conclusions

In conclusion, we have presented the operation of an intrinsic, simple, low cost, and stable displacement sensor based on a single fiber-loop made with a tapered fiber. The operation of the device relies on the interaction between the fundamental and higher order cladding modes excited controlled by the bend asymmetry in the refractive index profile induced by the bending, which depends on the curvature radius of the loop. In contrast with other schemes based on bending tapered fibers, the interaction zone is not perturbed during the displacement measurement. The proposed device allows the interrogation by several methods including the spectral measurement of the wavelength shift of the transmission notches, the measurement of the variations of the transmitted power, or dual wavelength referencing. In a displacement range of 3.5 mm, we have measured ∼360.93 nm of wavelength shift of one of the spectral notches, which means ∼0.116 nm/μm of sensitivity. On the other hand, from intensity based measurements using a low power LED source with 100 nm bandwidth and a Ge photodetector, we obtained a maximum sensitivity in the order of 2.7 nW/m in ∼1 mm displacement range. In both cases, the displacement resolution is in the order of microns, being susceptible of improvement by using narrowband light sources, polarimetric schemes, or higher sensitivity spectral analyzers or photodetectors.
